# Impact of Running on the Risk and Progression of Knee Osteoarthritis: A Review of Current Evidence

**DOI:** 10.7759/cureus.112416

**Published:** 2026-07-10

**Authors:** Michal Glinski, Urszula Kacprzak, Dominik Dolega-Mostowska, Natalia Adamaszek, Laura Stochaj, Julia Sochowska, Agnieszka Buczkowska, Julia Danieluk, Michalina Flejszman, Justyna Wójcik

**Affiliations:** 1 Hypertension, Internal Medicine, and Metabolic Disorders, Uniwersytecki Szpital Kliniczny w Poznaniu, Poznań, POL; 2 Internal Medicine, Uniwersytecki Szpital Kliniczny w Poznaniu, Poznań, POL; 3 Medicine, Uniwersytet Medyczny w Łodzi, Łódź, POL; 4 Paediatrics, Uniwersytecki Szpital Kliniczny w Poznaniu, Poznań, POL; 5 Internal Medicine, Diabetology, Cardiology, and Nephrology, Szpital Wojewódzki w Poznaniu, Poznań, POL; 6 Pulmonology, Allergology, and Pulmonary Oncology, Uniwersytecki Szpital Kliniczny w Poznaniu, Poznań, POL; 7 Endocrinology, Metabolism, and Internal Medicine, Uniwersytecki Szpital Kliniczny w Poznaniu, Poznań, POL; 8 Medicine, Uniwersytet Przyrodniczy w Poznaniu, Poznań, POL

**Keywords:** biomechanical window, chondroprotection, exercise programming, knee osteoarthritis, mechanobiology, recreational running

## Abstract

This review evaluates the safety of recreational running for knee health, defining the “biomechanical window” based on the latest evidence (2020-2024). The available evidence reveals a U-shaped risk curve, indicating that moderate recreational running is highly protective against knee osteoarthritis (KOA) compared with both sedentary lifestyles and elite, high-volume running. In patients over 50 years of age with existing OA, running does not accelerate structural damage and effectively reduces pain when managed through gradual load progression (the “10% rule”). Recreational running is a safe, chondroprotective intervention. Clinical guidelines should favour individualized load titration over the unfounded discouragement of activity.

## Introduction and background

Osteoarthritis (OA) currently represents an escalating challenge for global healthcare systems, as one of the most prevalent musculoskeletal conditions and a primary cause of disability among adults [1,2]. The prevalence of OA continues to rise globally, driven primarily by an aging population and the growing obesity epidemic [3]. Consequently, the increasing incidence of OA translates into a substantial socioeconomic burden on healthcare systems worldwide, affecting millions of individuals and significantly reducing their functional independence [1,4]. Knee cartilage damage and the subsequent development of OA lead to a profound deterioration in patients' quality of life, particularly in the psychological and social domains, which is strongly correlated with pain intensity [5]. Furthermore, recent epidemiological data highlight that joint cartilage injuries are increasingly diagnosed in younger, physically active populations, challenging the traditional view of OA strictly as a disease of advanced age [5].

Within this context, running - as one of the most accessible and popular forms of physical activity, with over 30 million marathon participants annually - remains a subject of controversy among both the general public and clinicians [6,7]. A so-called "running paradox" emerges: despite the fact that this activity generates ground reaction forces exceeding eight times body weight and is associated with a high incidence of overuse injuries, numerous epidemiological studies indicate a neutral or even protective effect of running on articular cartilage [8,9]. While the traditional "wear and tear" model perceived cartilage as a passive structure undergoing simple mechanical wear, modern evidence indicates that OA is a multifaceted disease resulting from a complex interaction between biomechanical loading, inflammation, genetics, metabolic health, and previous injury [10].

Crucial to the understanding of this relationship is the introduction of a clear dichotomy regarding exercise intensity. Scientific literature distinguishes recreational running (leisure/amateur), typically defined as activity undertaken for health-related purposes at a moderate volume (e.g., below 40-50 km per week), from elite/competitive running, which encompasses professional athletes, international competitors, and individuals subjecting their joints to extreme loads (exceeding 92 km per week) [11]. The foundation of modern knowledge regarding joint health in runners is the concept of mechanotransduction. Living articular cartilage possesses the capacity for conditioning; molecular processes occurring within chondrocytes, modulated by mechanoreceptors (such as the α5β1 integrin), facilitate the adaptation of the extracellular matrix to cyclic loading, thereby enhancing its resistance to damage [9,12,13].

This review aims to synthesize evidence from the past five years to determine whether running is safe for patients with OA and which exercise dosages define the "biomechanical window" for joint health [14].

## Review

Research materials and methods 

The present narrative review was developed based on a comprehensive analysis of relevant scientific literature, including clinical trials, meta-analyses, and systematic reviews. The selection process prioritized publications from 2020 to 2024 to reflect the most recent paradigms in sports medicine. Emphasis was placed on studies utilizing the Mendelian Randomization (MR) method, which allows for the assessment of the causal relationship between physical activity and OA while minimizing bias stemming from confounding factors [15]. Furthermore, the analysis incorporated large individual participant data (IPD) meta-analyses involving cohorts exceeding 5,000 participants [16], as well as systematic reviews of imaging studies (MRI) evaluating transient and long-term changes in cartilage morphology [8,10]. The research material encompassed diverse populations, ranging from sedentary individuals to elite ultramarathoners, and included varied joint health statuses, such as healthy individuals, high-risk groups following anterior cruciate ligament (ACL) injuries, and patients with an established diagnosis of OA [11,17,18]. The synthesis of data focused on integrating findings from studies providing a high level of clinical and mechanistic evidence to map the current state of knowledge.

Genetic and epidemiological evidence of causality: a 2024 perspective 

A breakthrough in understanding the relationship between physical activity and knee OA (KOA) has been provided by MR studies. The work by Ma et al. [15] currently represents the most robust genetic evidence for a causal impact of various activity levels on the risk of KOA. The researchers utilized single nucleotide polymorphisms (SNPs) as genetic instruments, analyzing data from the UK Biobank (N = 403,124 cases of KOA).

The results of this study indicate the existence of a curvilinear (U-shaped) relationship: (i) sedentary behavior: a strong causal association with an increased risk of KOA was demonstrated. Individuals genetically predisposed to a sedentary lifestyle (<1.5 METs) had more than twice the odds of developing the disease (OR 2.10; 95% CI: 1.51-2.92; p = 1.14 × 10⁻⁵) [15]. This mechanism is explained by the atrophy of joint-stabilizing muscles and the weakening of cartilage metabolism, which requires the “pumping” of synovial fluid during movement to receive adequate nutrition [9,15]. (ii) Appropriate physical exercise: defined as activity between 1.5 and 5.9 METs, this proved to be a significant protective factor. A genetic inclination toward regular moderate activity was associated with a drastic reduction in KOA risk (OR 0.15; 95% CI: 0.04-0.58; p = 0.006) [15]. (iii) Excessive physical activity: high-intensity activity (≥6 METs), characteristic of competitive sports, was identified as a risk factor, increasing the probability of KOA more than twofold (OR 2.16; 95% CI: 1.33-3.52; p = 0.002) [15]. These data are consistent with the IPD meta-analysis conducted by Gates et al. [16], which, based on six cohorts (5,065 participants) followed for 5-12 years, found no association between recreational physical activity and incident symptomatic OA. This suggests that, for the general population, recreational running falls within a safe physiological margin [11,16]. 

Dichotomy of OA prevalence: amateurs vs. competitive runners vs. control group

Critical data regarding the prevalence of OA in runners are provided by the systematic review and meta-analysis by Alentorn-Geli et al. [11], which included 125,810 individuals. This study enabled a precise comparison of OA prevalence based on running intensity. (i) Recreational runners (amateurs): this group demonstrates the lowest prevalence of hip and KOA, at only 3.5%. Significantly, the risk of developing OA in amateurs is substantially lower than that in the control group [2,11]. (ii) Sedentary controls: in the population leading a sedentary lifestyle, the prevalence of OA is 10.2%. This indicates that a lack of joint loading is nearly three times more detrimental than regular recreational running [11,15]. (iii) Competitive runners (elite): among elite athletes, OA prevalence increases to 13.3%. This risk is particularly high for individuals with a history of international competition (e.g., the Olympic Games) and those maintaining volumes exceeding 92 km per week for many years [11].

Modern studies of amateur marathon runners corroborate these observations. In a study by Hartwell et al. [19] involving a cohort of 3,804 Chicago Marathon participants, the prevalence of OA was found to be 7.3%, a figure significantly lower than the average for the general population in the same age group (approximately 26% for those over 45 years of age). Furthermore, the number of completed marathons, running history (cumulative years), and running pace were not significant predictors of OA development, whereas age, BMI, and previous injuries played decisive roles [19]. This dichotomy suggests that recreational running serves a chondroprotective function, and the issue of "wear and tear" pertains exclusively to extreme training volumes combined with inadequate recovery [9,11]. 

Biomechanical mechanisms: cyclic loading and cartilage adaptation 

A fundamental element in understanding the influence of running on the joints is resolving the so-called "loading paradox." Despite generating peak reaction forces significantly higher than those of walking, recreational runners do not exhibit a higher prevalence of OA [9,11,20]. The explanation for this phenomenon rests on the Cumulative Load Theory [9,20]. According to this theory, OA risk is more strongly correlated with the cumulative load per unit of distance than with the peak force of a single step. Runners take fewer steps per kilometer due to longer flight phases and greater stride lengths, and their ground contact duration is significantly shorter [9,20]. As a result, the averaged cumulative load accumulated during a one-mile run is comparable to - and in some models, even lower than - the load accumulated during walking the same distance [20]. Additionally, the viscoelastic properties of cartilage ensure that its response to loading depends on the loading rate. The high loading rates associated with running increase the dynamic stiffness of the cartilage, which limits internal deformations (strains), thereby protecting the collagen network from excessive stretching [9]. Modern imaging studies utilizing high-field magnetic resonance (3T MRI) confirm that cartilage responds to running in a physiological rather than pathological manner. The meta-analysis by Coburn et al. [10] and the review by Khan et al. [8] indicate the occurrence of transient cartilage deformation immediately following exercise. A reduction in knee cartilage volume and thickness of approximately 3.3%-4.9% has been demonstrated immediately post-run [10]. These changes, also visible in T2 mapping (shortening of relaxation time), result from the transient outflow of water from the extracellular matrix into the joint space [8,10]. Most significantly, cartilage in healthy individuals exhibits the capacity for rapid regeneration, returning to baseline parameters within 1 to 24 hours after the cessation of running [10,21]. The absence of new structural damage formation even after a marathon suggests that cyclic mechanical loading remains within the biological tolerance limits of the joint [7,8]. 

Biochemical and molecular mechanisms: the role of markers and cytokines

At the molecular level, the pathogenesis of OA involves chronic oxidative stress, where an accumulation of reactive oxygen species (ROS) inactivates antioxidants and exacerbates cartilage matrix degradation through the upregulation of degrading proteases [22]. Conversely, regular recreational running can actively mitigate these catabolic pathways by lowering intra-articular pro-inflammatory cytokines and facilitating the transport of structural debris out of the joint space [3]. The impact of running on joint metabolism is measurable through the analysis of biomarkers in serum and synovial fluid. A key indicator is sCOMP (serum cartilage oligomeric matrix protein), an extracellular matrix protein whose serum concentration transiently increases post-run [13,23]. While COMP was formerly recognized as a marker of damage, sCOMP is now perceived as an indicator of active cartilage metabolism and tissue turnover [13]. Research by Hyldahl et al. [24] demonstrated that while serum COMP concentrations rise after a 30-minute run, intra-articular concentrations in the synovial fluid decrease. This suggests a mechanical "wash-out" of metabolic products from the joint into the bloodstream, which facilitates their elimination and promotes matrix renewal [3,13,24]. Running also exhibits significant anti-inflammatory effects. It has been shown that regular activity of moderate intensity reduces the concentration of pro-inflammatory cytokines, such as IL-1β and TNF-α, within the joint [12,24]. Simultaneously, it promotes the release of chondroprotective cytokines, such as interleukin-10 (IL-10) [12,25]. This anti-catabolic response is strongly supported by mechanotransduction pathways that suppress the secretion of matrix metalloproteinases (such as MMP-13), directly counteracting cartilage degradation and chondrocyte apoptosis [12]. Concurrently, mechanical joint stimulation during running increases lubricin production by type B synoviocytes, which improves joint tribology (lubrication) and reduces surface friction [12,13]. Recent genetic and molecular evidence further elucidates these adaptive responses, demonstrating that moderate cyclic loading upregulates the expression of lncRNA H19, which acts as a crucial molecular buffer mitigating post-traumatic inflammatory pathways and cartilage degeneration [14]. Moreover, long-distance endurance training induces profound systemic metabolic adaptations that support overall chondrocyte resilience, particularly through enhanced fuel substrate catabolism and protective micronutrient signaling [26]. 

Running in patients with pre-existing OA

Traditional medical recommendations often discouraged patients with OA from running, fearing accelerated structural progression. However, recent evidence, including the seminal study by Lo et al. [27] based on data from the Osteoarthritis Initiative (OAI), challenges this paradigm. Analysis of a cohort of individuals over 50 years of age with radiographic KOA demonstrated that running does not increase the risk of symptom exacerbation or structural progression (measured by Joint Space Width/Narrowing) over a 48-month period [2,27]. Furthermore, runners with OA reported significant pain reduction (OR for knee pain resolution = 1.7) compared with non-runners [27]. This mechanism may stem from the analgesic effect induced by muscle stimulation and improved proprioception [27]. Running in patients with OA leads to subjective improvement in health-related quality of life (HRQoL) and physical function, provided that training volume is adjusted to current clinical symptoms [28,29]. These findings suggest that, in patients with OA, recreational running should be considered a safe form of therapy rather than a detrimental factor [4,12,27]. For patients whose symptoms progress and conservative treatments - such as intra-articular platelet-rich plasma (PRP) or bone marrow aspirate concentrate (BMAC) injections - fail to provide adequate pain relief, total joint arthroplasty remains the definitive therapeutic option to restore mobility and joint function [28]. Nonetheless, appropriately tailored recreational running, defined as distances up to 25 miles per week, is currently advocated as a safe, non-pharmacological strategy that does not accelerate structural OA progression and effectively improves clinical outcomes in properly selected individuals [2,3]. 

Interpretation of the "biomechanical window": the golden mean hypothesis

Synthesis of the collected evidence, particularly the most recent MR studies, allows for the formulation of the "biomechanical window" concept for knee joint health [9,15]. Genetic analysis results indicate a non-linear (U-shaped) relationship between activity levels and OA risk [15]. On one hand, sedentary behaviors are associated with a more than twofold increase in the risk of KOA (OR 2.10), which is attributed to a lack of cartilage conditioning and the weakening of joint metabolism, which necessitates the cyclic “pumping” of synovial fluid into avascular tissue [9,15]. On the other hand, excessive physical activity (≥6 METs), characteristic of competitive sports, also significantly increases the risk (OR 2.16) [15]. Between these extremes lies an optimal activity window (1.5-5.9 METs), which demonstrates a strong protective effect (OR 0.15) [15]. Recreational running fits perfectly within this chondroprotective margin, promoting mechanical adaptation without exceeding the threshold of biological tissue tolerance [6,11]. 

Modifying factors: BMI, injury, and age 

Analysis of the evidence indicates that it is not the act of running itself, but rather the accompanying factors that determine joint health in runners [6,19]. BMI remains a key modifier - each unit increase in BMI is associated with a 10% increase in the probability of developing OA (OR 1.10) [19]. An even stronger predictor is previous joint injuries and surgeries, such as ACL reconstruction or meniscectomy, which increase OA risk more than fivefold (OR 5.04-5.85) [19]. These injuries permanently alter joint biomechanics and can lead to secondary OA (post-traumatic OA, PTOA) even under moderate loading [14,17]. Age, while naturally correlated with the progression of degenerative changes (OR 1.08 per year of life), does not preclude running - individuals over 50 years of age can safely continue this activity, provided they control other risk factors [19,27]. Ultimately, physical activity acts as a double-edged sword for joint health; while moderate endurance training preserves cartilage integrity, excessive high-impact running can accelerate joint degeneration if not counterbalanced by sufficient recovery [6]. Despite these potential risks, appropriately balanced marathon training is associated with a significantly lower prevalence of hip and KOA (8.8%) compared with the general population (17.9%) [26]. Therefore, the safety of long-term running is highly individualized and strongly mediated by predisposing risk factors, highlighting the need for personalized, patient-specific exercise recommendations [6]. 

Environmental impact: air pollution (PM2.5)

A novel theme in the literature is the impact of air pollution on the aggravation of OA severity in active individuals [22]. Exposure to particulate matter (PM2.5) during exercise may exacerbate OA through mechanisms of systemic inflammation [22]. It has been demonstrated that PM2.5 stimulates the release of pro-inflammatory cytokines (TNF-α, IL-6, and IL-1β) and the polarization of M1 macrophages in the lungs, which translates into an increased degradation of the synovial matrix and a decrease in subchondral bone mineral density in the knee joints [22]. This suggests that, to optimize joint health, runners should avoid areas with high concentrations of smog [22]. 

Practical clinical recommendations 

Key clinical recommendations emphasize the necessity of a structured, individualized approach to load management, specifically through the gradual progression of training loads. Because articular cartilage exhibits viscoelastic properties and requires time to undergo mechanobiological adaptation, sudden spikes in training volume or intensity can overwhelm the tissue's remodeling capacity [9]. The “10% rule” is a widely accepted guideline used in clinical and recreational settings, aimed at reducing the risk of injury by limiting the weekly increase in running volume or intensity to a fixed level of 10%. Despite the widespread use of this rule as a practical strategy for safely adjusting mechanical load, its effectiveness is not robustly supported by evidence from high-quality clinical studies. Consequently, the most recent literature emphasizes that effective injury prevention and the establishment of a safe “biomechanical window” likely require a more individualized approach to load management rather than a one-size-fits-all approach based on percentage values [29]. Furthermore, diligent symptom monitoring is paramount for establishing a safe biomechanical window for each patient. Clinicians should educate patients to distinguish between benign, transient discomfort and signs of joint overload. The application of the "24-hour rule" is a widely recognized standard in rheumatology and sports medicine: if knee pain, swelling, or stiffness exacerbates during or immediately after a run and persists for more than 24 hours, it indicates that the applied mechanical load has exceeded the joint's current biological tolerance [29]. Crucially, the manifestation of such symptoms should not automatically lead to the complete cessation of running. Instead, it should serve as a clinical signal to proactively modify training parameters. Evidence suggests that adjusting specific variables - such as reducing the total running distance, decreasing running speed, lowering the weekly frequency of runs, or increasing recovery time between sessions - is a highly effective strategy to manage symptoms while maintaining the chondroprotective benefits of cyclic loading [21]. Complete rest is rarely warranted; rather, a temporary reduction in load, followed by a gradual reintroduction of running, allows the joint to recover and adapt safely without progressing the structural damage of OA [21,29]. 

Limitations of current knowledge

It must be acknowledged that a significant portion of the literature is based on retrospective studies and surveys, which entail a risk of recall bias [6,11,19]. Many meta-analyses exhibit high heterogeneity due to inconsistent definitions of "competitive" versus "recreational" running [11,29]. Additionally, the "healthy runner" effect (selection bias) may cause individuals with early pain symptoms to quit running before being enrolled in studies, potentially resulting in an apparent but overestimated protective effect of physical activity on structural outcomes [9,27]. Also, many of the cited cross-sectional or retrospective designs do not accurately track lifetime cumulative running exposure, making it difficult to precisely isolate the long-term impacts of running from other confounding lifestyle factors. Consequently, recent comprehensive reviews emphasize the critical need for robust, longitudinal prospective studies to definitively validate the parameters of this chondroprotective window and establish long-term clinical guidelines [6,10,14]. 

## Conclusions

Current evidence suggests that recreational running is not associated with an increased risk of KOA in most healthy individuals and, indeed, demonstrates a protective effect, resulting in a significantly lower prevalence of OA (3.5%) compared with sedentary populations (10.2%). Moderate mechanical loading stimulates the secretion of lubricin and reduces the levels of intra-articular pro-inflammatory cytokines, thereby promoting cartilage homeostasis. In patients over 50 years of age with radiographic KOA, running does not accelerate structural progression (measured by Joint Space Width) and may lead to a significant reduction in knee pain. The risk of OA increases significantly only under extreme competitive loading conditions (>92 km/week) or in the presence of untreated structural injuries. There is a critical necessity to conduct long-term prospective cohort studies utilizing advanced qMRI imaging, including T2 mapping, to detect subclinical changes in cartilage hydration and composition.

Future research should also focus on investigating the long-term trajectory of joint health in runners following ACL reconstruction (e.g., the TRAIL project) to develop precise return-to-sport guidelines that minimize the risk of PTOA.
